# Blood Pressure Awareness and Knowledge of Cardio-Cerebrovascular Diseases in South Korean Women with Hypertension

**DOI:** 10.3390/healthcare9030360

**Published:** 2021-03-23

**Authors:** Yeo Won Jeong

**Affiliations:** Department of Nursing, College of Medicine, Dongguk University, Gyeongju 38066, Korea; ywjeong@dongguk.ac.kr

**Keywords:** cerebrovascular disorder, stroke, hypertension, women, knowledge, awareness

## Abstract

(1) Background: It is essential to increase the awareness of cardiovascular diseases’ symptoms and reduce treatment delays among women with hypertension (HTN). This study aimed to assess the knowledge of cardio-cerebrovascular diseases’ warning signs (KCVDs), according to awareness of their blood pressure levels (AoBP), and identify the factors associated with KCVDs and AoBP in women with HTN. (2) Methods: This study used the data from the Korea Community Health Survey of 2018. A total of 29,832 women with HTN were included in the final analysis. Data on sociodemographic characteristics, KCVDs, and AoBP were identified. A negative binomial regression was used to identify factors associated with KCVDs. (3) Results: Of the participants, 42.9% were not aware of their blood pressure level, and 9.1% did not have any knowledge of KCVD symptoms. Factors associated with KCVDs were AoBP (odds ratio (OR) = 1.121, *p* < 0.001), middle age (OR = 1.012, *p* = 0.008), employment (OR = 1.034, *p* < 0.017), and being married and living with a spouse (OR = 1.068, *p* < 0.001). Lower levels of education (OR = 0.931, *p* < 0.001) and regular walking (OR = 0.964, *p* = 0.015) were also associated with KCVDs. Health-related quality of life (HRQoL) and subjective health status were associated with increased AoBP. (4) Conclusions: AoBP was positively associated with KCVDs. It is necessary to include AoBP in public campaigns and regular policy support to improve KCVDs. In addition, findings in this study can serve as basic data for developing socio-cultural interventions, aimed at mitigating cardio-cerebrovascular diseases, by improving levels of KCVDs.

## 1. Introduction

Cardiovascular diseases (CVDs) are a leading cause of death worldwide [1,2,3]. In South Korea, they are the second-highest cause of death after neoplasms. In 2018, the mortality rate from CVDs was 122.7 per 100,000 people, which showed a 10.4% increase from 2008 [4]. In particular, the prevalence of hypertension as a risk factor of CVDs in women has increased steadily over the past 10 years [5], and women (122.7) had a higher mortality rate from CVDs than men (112.0) in South Korea in 2019 [6]. 

CVDs are heart and blood vessel disorders and include cerebrovascular diseases [3]. Heart attack and stroke are representative diseases of CVDs [3,7]. Many studies reported that women were more likely to experience a higher lifetime incidence of CVDs than men, because of longer average life expectancy, menopause, and increased prevalence of CVD risk factors such as HTN, obesity, and diabetes mellitus (DM) [1,2,8]. In addition, when CVD symptoms occur, rapid hospitalization and immediate treatment are the most important factors in determining the patient’s prognosis [9,10]. Treatment within the first 60 min of the stroke onset is associated with the best outcome and early hospital discharge [9]. Additionally, the goal of treatment in ST-segment elevation myocardial infarction is to achieve reperfusion within 120 min of onset [11]. Despite the importance of visiting a hospital for rapid treatment in South Korea, the delay rate of emergency medical centers within the ideal timeframe (within 2 h) after acute myocardial infarction onset was 56.0%, and 1.35 times higher in women than men [12]. The main reason for delayed hospital arrival in women was a lack of warning sign recognition and understanding of atypical symptoms without typical symptoms such as chest pain [12,13,14]. In this regard, Woodward referred to the female disadvantage [8]. Woodward mentioned that atypical symptoms are attacks often experienced by women in a different way compared to men, which may lead physicians to misdiagnose heart attacks because classic medical text-book models were written according to the male experience of the disease [8]. To minimize complications and reduce mortality rates due to CVDs, prompt and appropriate treatment should be administered, and it is important to know CVDs’ warning signs. However, previous studies showed that less than half the people (15.6% to 48.8%) were aware of all the warning symptoms [15,16,17,18]. As mentioned above, women are more prone to lack recognition of knowledge of CVDs warning signs (KCVDs) because of experiencing atypical symptoms; this was reported as the cause of increased treatment delay among women [12,13]. Considering the increased prevalence of HTN, a prominent risk factor for CVDs, among South Korean women [5], it is necessary to evaluate the level of KCVDs and its associated factors in women with HTN.

The factors associated with KCVDs were age, residence, marital status, education level, income, smoking, alcohol consumption, body mass index (BMI)/obesity, comorbidities such as DM, (family) history of CVDs, and exercise [1,2,8,15,17,18,19]. Concerning mental health, health-related quality of life (HRQoL), depression, and subjective health status are associated with HTN [20,21,22]. Urban HTN patients may have a higher HRQoL [20] and depression is an independent risk factor for HTN [21]. Subjective health status is also associated with HTN [22]. Since the target population was adult women with HTN, based on previous studies, it is necessary to include mental health factors affecting HTN. 

Increased BP levels are associated with an increased risk of developing CVDs [3]. In HTN patients, when the BP level exceeded 150 mmHg (versus > 120 mmHg), it increased the first ischemic stroke incidence by 1.6 times [23]. Additionally, stroke incidence was 1.8 times higher in hypertensive patients who never had a BP measurement throughout their lifetime [24]. Patients in Asia did not view HTN as a serious condition and ignored high BP because HTN has no overt physical symptoms [25]. Thus, awareness of their BP level (AoBP) is more likely to detect uncontrolled BP and might influence health-related interests such as KCVDs. To the best of our knowledge, no study has been conducted on this issue in South Korea. In addition, some studies have reported the awareness of CVD warning signs using the Korean Community Health Survey (KCHS) data [17,18,19,26]; however, no study has investigated the warning signs of heart attack as well as stroke, while targeting women with HTN, as one of the risk factors of CVDs. Therefore, this study aimed to evaluate the level of KCVDs, according to AoBP, in women with HTN, and the associated factors with KCVDs and AoBP.

## 2. Methods

### 2.1. Participants and Data Collection

This cross-sectional study used the KCHS data collected from women diagnosed with HTN, between August and October 2018 [27]. It is a large-scale sample survey conducted annually by the Korean Center for Disease Control and Prevention for adults aged 19 years or older. It was performed through computer-assisted personal interviews conducted by trained interviewers. The height and weight measurements of the participants were conducted starting in 2018. The sampling procedures of the KCHS had two stages: (1) the primary sample unit was extracted proportionally based on the number of households by housing type in the resident area (street/village/district) using the extraction probability method considering household size, and (2) the number of households was identified in the selected sample and then extracted through systematic sampling methods. In 2018, the questionnaire had 21 categories and 201 questions on demographics, socioeconomic characteristics, and physical and psychological health. Data were collected by trained investigators, who visited each household, explained the purpose of the KCHS to the participants, and then interviewed them using a notebook, during face-to-face interviews, after obtaining written consent. The sampling procedures, participants, variables, and weights are reported on the KCHS website [28]. Of the 228,340 individuals who completed the KCHS in 2018, the following were excluded: (1) men (*n* = 102,241), (2) women who were not medically diagnosed with HTN (*n* = 89,107), and (3) those with missing data (*n* = 7160) about the main variables (AoBP and knowledge of CVDs). Therefore, a total of 29,832 women, who were medically diagnosed with HTN, were included in the analysis.

### 2.2. Variables

#### 2.2.1. Knowledge of CVDs’ Warning Signs and Awareness of Their Blood Pressure Level

To measure knowledge of CVDs, the Behavior Risk Factor Surveillance System Questionnaire published in 2009 by the Center for Disease Control and Prevention [29] was translated by the Korean Disease and Prevention Agency and professionals at regional cardiovascular disease centers at nine university hospitals and modified according to the South Korean context. Of the 12 items, 10 related to heart attack and stroke warning signs were used. The correct answer to each item was one point, and incorrect answers or not knowing was treated as zero points [27]. The score was the number of correct answers, and the total score was distributed between 0 and 10 points. This tool’s KR-20 (Kuder Richardson formula) in another study was 0.85 [30], and 0.908 in this study. AoBP was assessed through the question, “Do you know your blood pressure level?” Responses to each item were either yes or no.

#### 2.2.2. Health-Related Quality of Life and Subjective Health Status

To measure HRQoL, the Korean version of the EuroQol-five-dimensions three-level version (EQ-5D-3L) [31] developed by the EuroQol group [32], was used. The EQ-5D-3L has five dimensions: mobility, self-care, usual activities, pain or discomfort, and anxiety or depression, with three different levels: 1, no problem; 2, some problems; and 3, severe problems. The EQ-5D-3L index was calculated using the Korean value set of the Korean version of the EQ-5D-3L, where higher scores indicated better overall health status [33]. In this study, the Cronbach’s alpha for the EQ-5D-3L was 0.817. 

To measure subjective health status, participants were asked, “What do you usually think of your health?” rated on a five-point scale (very poor, poor, average, good, very good) as self-rated health, which was consistent with objective health status [34].

#### 2.2.3. Depression

Depression was assessed using the Korean version of PHQ-9 [35], developed by Spitzer, Kroenke, and Williams [36]. This scale comprises nine items rated 0–3 points each and a total score ranging from 0–27. Higher scores indicate higher levels of depression. Cronbach’s alpha was 0.844 in this study.

#### 2.2.4. General Characteristics

Socio-demographic variables included age, education, monthly income, occupation, residence location, marital status, smoking, alcohol consumption, obesity, walking exercise, and comorbidity. Ages were divided into 19–39, 40–64, and 65 years or older, in accordance with previous studies [37]. Education was classified as high school graduation or lower. The monthly average household income was classified as less than two million Korean won. Occupation was divided into two groups: currently employed or unemployed. Residence location was classified as rural if the respondent lived in a town/township and as urban if the respondent lived in a city neighborhood. Marital status was divided into married and living with a spouse or others, including divorced, separated, widowed, or never married. Smoking status was assessed using the item, “Are you smoking now?” Responses to this item were used to categorize participants as non-smokers (including past smokers) and current smokers. Alcohol consumption was assessed using the item, “Did you drink alcohol in the last year?” Responses to the items were either yes or no. Obesity was assessed using the body mass index (BMI, kg/m^2^), which was measured by the trained investigators, in accordance with the Asia-Pacific region criteria [38] and divided into obese (BMI ≥ 25.0) and normal. Walking exercise was assessed using the item, “How many days have you walked at least 10 min at a time in the last week?”, and responses were divided into three groups: <30 min, one day per week or less; <30 min, two to four days per week; >30 min, five days per week or more. HTN management was assessed as using HTN medication. DM was also defined as a comorbidity by a medical doctor. 

### 2.3. Statistical Analysis

**Figure 1:**
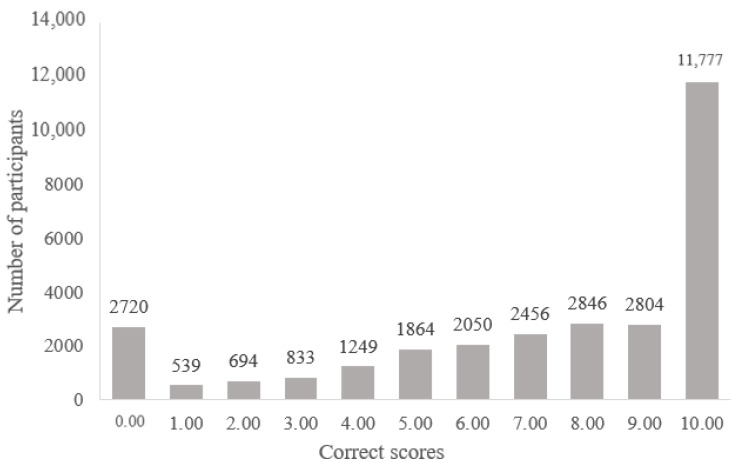
Scores for knowledge about warning signs of cardio-cerebrovascular diseases.

Data were analyzed using SPSS 22.0 (IBM Corp., Armonk, NY, USA). General characteristics and good KCVDs are presented as frequencies and percentages. Depression, sleep quality, HRQoL, and level of KCVDs were presented as means and standard errors. In addition, *t*-test and Chi-square tests were used to analyze the differences between KCVDs and psychological factors, according to AoBP. The continuous variables showed a range of skewness and kurtosis, from −2.125 to 2.257 and from −0.415 to 7.023; therefore, the effect size was calculated by Cohen’s d and Cramer’s V. Participants with four or five correct answers for each CVDs’ warning sign were classified as having good KCVDs, and those with three or fewer as having poor KCVDs, based on previous studies [15,19]. Negative binomial regression (NBR) was performed to identify the factors associated with KCVDs because (1) the variance (10.83) of the dependent variable, the KCVDs score was larger than the mean (7.17), and (2) the KCVDs score was skewed at both ends (Figure 1) [39,40]. The suitability of the model coefficients for the NBR model was verified using the likelihood-ratio test. As a result, it was found that the probability of significance in the likelihood-ratio test was less than 0.001, so the data explained the model well. The statistical significance was set at *p* < 0.05.

## 3. Results

### 3.1. General Characteristics According to AoBP

Of the 29,832 women HTN participants, 42.9% did not know their BP level, and among them, 10,202 (79.7%) were over 65 years old. Of the participants who knew their BP (the awareness group), a higher proportion were living in urban areas (52.1%) than rural areas (47.9); were married and living with their spouse (65.2%) than those who were divorced, separated, widowed, or never married (34.8%); and walked more than five days per week (50.5%) compared to those who walked less than four days per week. In contrast, the non-awareness group showed a higher rate of living in rural areas (64.3%) than urban areas (35.7%); were divorced, separated, widowed, or never married (53.8%) compared to those who were married and living with their spouse 46.2%); walked once or less in a week (42.9%). In both groups, the proportion of those with less than high school education, less than two million monthly income, unemployed, non-smoker, obese, consumed alcohol during the previous year, and taking a medication for HTN was higher. Of all participants, 7128 (23.9%) had DM (Table 1).

### 3.2. Level of KCVDs in Adult Women with Hypertension

Of all participants, 9.1% (*n* = 2720) could not identify the 10 KCVDs items (Figure 1). The percentage who never identified heart attack or stroke warning signs was 11.8% (*n* = 3521) and 12.6% (*n* = 3754), respectively. In addition, participants who identified all of the heart attack and stroke, or only heart attack or stroke items were 39.5% (*n* = 11,777), 52.4% (*n* = 15,637), and 46.0% (*n* = 13,714) respectively. As shown in Table 2, the good KCVDs were higher in the awareness group (χ^2^ = 701.172, *p* < 0.001), although effect size was weak (Cramer’s V = 0.15). The awareness group had significantly higher subjective health status (*t* = −15.443, *p* < 0.001, Cohen’s d = 0.29), lower depressive symptoms (*t* = −9.752, *p* < 0.001, Cohen’s d = −0.19), and higher HRQoL (*t* = 24.70, *p* < 0.001, Cohen’s d = 0.35). 

**Figure 2:**
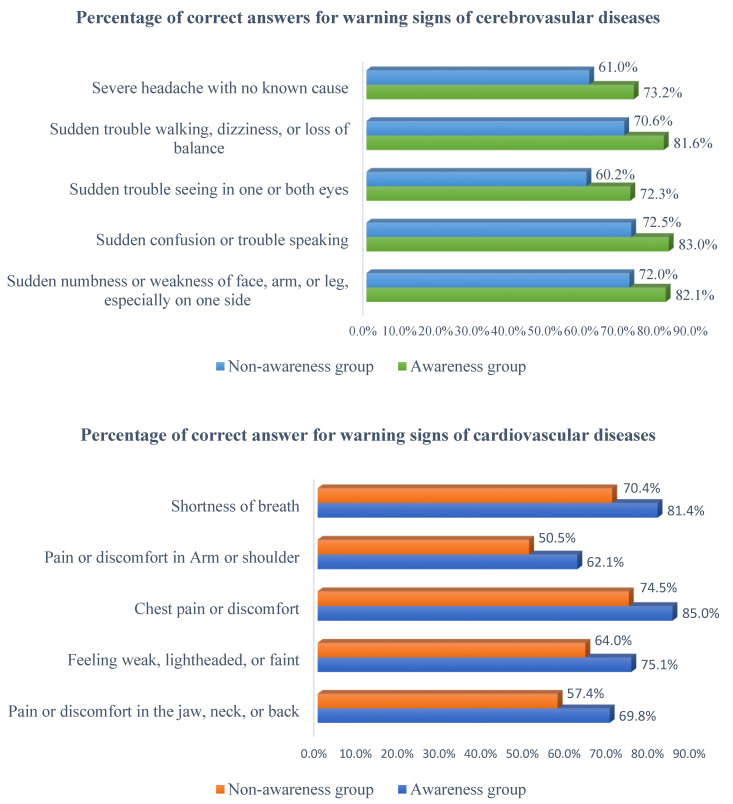
Percentage of participants who recognized each symptom of the Knowledge of CVDs warning signs.

The percentage of correct answers on the 10 items measuring CVDs knowledge was higher in the awareness group (Figure 2). Among the five items on cerebrovascular disease, the item “sudden trouble walking, dizziness, or loss of balance” was the item with the lowest score in both groups. The item “sudden confusion or trouble speaking” was the item with the highest score in both groups. Regarding cardiovascular disease knowledge, “pain or discomfort in arm or shoulder” was the item with the lowest score in both groups, and “chest pain or discomfort” was the item with the highest score in both groups.

### 3.3. Factors Associated with KCVDs and AoBP

Factors associated with KCVDs warning signs are listed in Table 3. Among women with HTN, more BP level awareness was associated with better KCVDs (OR = 1.121, *p* < 0.001). Middle-age (OR = 1.046, *p =* 0.008), employment (OR = 1.034, *p =* 0.017), and married and living with a spouse (OR = 1.068, *p* < 0.001) were associated with better KCVDs. In addition, lower levels of education (OR = 0.931, *p* < 0.001) and regular walking (OR = 0.964, *p* = 0.015) were associated with lower KCVDs. 

Factors associated with AoBP were middle-aged adults, high education, employment, living in urban areas, married and living with a spouse, obesity, regular exercise (walking two days per week or more), and taking medication for HTN (Table 4). Among women with HTN, the higher the subjective health status, HRQoL, and knowledge of CVDs the better the AoBP (OR = 1.095, *p* < 0.001, OR = 2.048, *p* < 0.001, OR = 1.076, *p* < 0.001, respectively).

## 4. Discussion

Among Korean female adult patients with HTN, 9.1% were not aware of any of the 10 warning signs of KCVDs, and 39.5% knew all 10 signs. A total of 52.4% and 46.0% knew all stroke and heart attack warning signs, respectively, suggesting they were less aware of the latter. There is no study on Korean adult female patients with HTN, which limits the direct comparison of our results. However, our result was 3.6% higher than those aware of heart attack warning signs in the 2017 KCHS [18], but it was still less than 50%. In particular, participants in our study were mostly aware of chest pain and shortness of breath, and less aware of signs such as feeling weak, lightheaded, or faint, pain or discomfort in the arm, shoulder, jaw, neck, or back. Studies in Kuwait [16], China [41], and nine European countries [42], showed the proportion of those aware of heart attack warning signs was lower than those aware of stroke signs and showed similar tendencies for symptoms. These findings suggested the general public has low awareness of faint or radiating pain caused by heart attacks. Patients do not experience all symptoms during a heart attack and may complain of syncope, weakness [12,13,43], or arm and shoulder pain or discomfort [44]. In particular, women reported milder symptoms [1] and complained more about back pain and shoulder blade pain or discomfort [17,44]. Also, atypical symptoms such as abdominal discomfort, fatigue, cough, fever, and nausea/vomiting were observed [1,12,13,43]. In our study, 11.8% of participants were unaware of any warning signs, and 52.4% were aware of all stroke signs. Although a direct comparison is difficult, 11.8% and 48.8% of diabetes patients with HTN were not aware of any warning signs and were aware of all signs, respectively, similar to our study. Also, a severe headache with no known cause and sudden trouble seeing in one or both eyes had the lowest awareness level as a warning sign of a stroke in our study, similar to other studies [16,17,19,41]. Stroke symptoms experienced by hospitalized patients include dizziness, dysarthria/slurred speech, hemiplegia, general weakness, facial paralysis, and consciousness disturbance, along with less common symptoms such as headache, nausea/vomiting, and convulsion [45,46]. Significantly, women experience atypical symptoms, including cognitive dysfunction, nausea/vomiting, headache [47], visual disturbance, and diplopia [48], which have low awareness. Warning signs and atypical symptoms of heart attack and stroke combined with low awareness were more common in women. This could lead to delayed treatment, which affects the patients’ prognosis. Not all CVDs are preceded by any warning signs. However, as HTN is a risk factor for CVDs, it is necessary to strengthen continuous education, publicity, and support for policies on CVDs’ warning signs for adult female patients with HTN. Moreover, education should emphasize that arm and shoulder pain or discomfort and fainting are heart attack warning signs, and a headache and visual impairment are stroke warning signs. In addition, women are more prone than men to delay treatment related to their CVDs because they may be more likely to put their family first [8]. This phenomenon is especially prominent in Asian women with oriental collectivistic values, who did not want to bother their family or financially burden their children, and thereby internally coped with CVD symptoms patiently [43]. Therefore, when educating women with HTN about KCVDs, it is necessary to encourage seeking immediate help from families or other close relatives for a better treatment outcome.

In our study, those with AoBP had significantly higher KCVDs than the non-awareness group. KCVDs increased by 1.121 times in those with BP awareness, and AoBP increased by 1.076 times as KCVDs increased, suggesting KCVDs and AoBP have a significant positive influence on each other. Additionally, the factors related to KCVDs were consistent with previous studies [16,17,18,19,24,26,41].

Among factors related to KCVDs in adult female patients with HTN, middle age, higher education level, employment, being married, living with a spouse, and regular walking exercise were associated with AoBP. BP level awareness increased HTN knowledge and interest, which promoted active participation in disease management and prevention of complications [49]. In particular, middle-aged women [50] with a higher level of education and occupation [51] were more likely to engage in health-promoting behaviors, and those with a higher education level were more likely to attempt health-promoting disease management behaviors [52]. Measuring BP was recommended for HTN management [53]. In our study, middle-aged women with HTN with a high education level and occupation showed high AoBP and performed more health-promoting behaviors. Furthermore, those who measured BP and recognized their BP was higher had a greater interaction level with the medical staff [53]. Consultation and interaction with medical staff acted as a source of CVDs knowledge [42], which increased knowledge and understanding because of a high education level. We observed those who lived with a spouse had an AoBP and KCVDs of 1.496 and 1.068 times, respectively. Previous studies showed those who live with a spouse measured their BP more often, increasing their AoBP [13,54], which is thought to contribute to KCVDs-related information. As AoBP increased, KCVDs increased in our study, and higher KCVDs decreased treatment delays, which can lead to a positive prognosis [13]. Therefore, it is necessary to emphasize measuring BP and education on warning signs. Furthermore, it would be important to encourage immediate medical staff consultation when BP is outside the normal range. We observed that living alone and low education were factors that delayed treatment in adult female patients with HTN when symptoms of CVDs appeared [14]. Therefore, education and policy support on KCVDs should ensure patients who have a low education level and live alone learn to make emergency calls, and visit the hospital via ambulance service immediately when warning signs are observed.

Unlike previous studies, this study showed that smoking and alcohol consumption were not related to KCVDs. This study included only adult female patients with HTN, whereas, in previous studies using KCHS, both men and women participated, and therefore, current smoking and risky/binge drinking were found to be factors influencing unawareness of warning signs of CVDs [17,19,26]. This inconsistency could be caused by the difference in the study sample. In Korea, men reported higher smoking and alcohol consumption rates, which were approximately 11 and 3 times higher, respectively, than women [26]. In our study, 98% and 53.2% of adult female patients, with HTN, were non-smokers and did not consume alcohol, respectively, in the last year. Therefore, it was believed that smoking and drinking were not factors influencing KCVDs in this study. However, smoking and drinking are risk factors for CVDs, and the former has an especially detrimental impact on CVDs among women than men [1]. Thus, it is necessary to provide education about warning signs of CVDs for current smokers and drinkers among this population along with tailored management for smoking cessation and restricted drinking.

Those prescribed with HTN medication had 1.437 times higher AoBP than those who were not prescribed. This may mean that those diagnosed with HTN and taking medication, measure their BP more frequently to assess the medication effects [53,55]. Additionally, high BMI, a risk factor for HTN, led to a higher BP measurement rate in a previous study [55], which is consistent with our study findings.

This study showed that the subjective health status and HRQoL were associated with AoBP. Additionally, depression was not related to AoBP and KCVDs; however, those with BP awareness showed significantly lower depression scores in the differential analysis. No previous study has assessed BP measurement or awareness and psychological variables including HRQoL in patients with HTN; thus, a direct comparison may be difficult. However, the purpose of measuring and being aware of BP levels, in women with HTN, is to prevent complications such as a stroke, and these actions increase confidence in controlling the BP [56], which helps elevate HRQoL [57] and subjective health status [58] and reduce depression [59]. The main variable of our study, AoBP, did not necessarily indicate that BP was controlled. However, based on factors that were associated with AoBP, the AoBP group was aware of their BP level and performed appropriate health-promoting behaviors, including taking medications, performing regular activities, and having high KCVDs. Additionally, factors that were associated with HRQoL in hypertensive patients in previous studies, such as living in urban areas, high education level, high physical activity, married and living with a spouse, and having a job [20], were observed in the AoBP group. These findings suggested AoBP did not directly increase HRQoL, but other socioeconomic characteristics and health behaviors interacted with and were associated with HRQoL.

The limitations of our study and future directions are as follows: (1) participants’ BP and how they became aware of BP were not assessed. Therefore, other factors, such as whether BP was controlled and self-monitored at home or measured at a medical institution were not considered. The author recommends evaluating the differences of KCVDs according to path aware of one’s BP level in future studies; (2) this was a secondary data analysis study, and only variables included in the original survey data were analyzed. Therefore, there were limitations in assessing and analyzing confounding factors related to BP or KCVDs, such as a history of stroke or heart attack in participants or family members and psychological variables including HRQoL and depression other than that of demographic factors. Future studies should consider these factors related to BP or KCVDs; and (3) awareness of heart attack and stroke warning signs was measured using closed-ended questions, which may have led to higher awareness. Despite these limitations, this study was the first to show the relationship between KCVDs and AoBP in adult women patients with HTN.

## 5. Conclusions

In South Korean women with HTN, KCVDs increased as AoBP increased, and KCVDs and AoBP varied as per age group and were influenced by employment, education level, marriage, living with a spouse, and regular exercise. Additionally, other factors associated with AoBP were urban residence, obesity, BP prescription medications, high subjective health level, and HRQoL. Women with HTN, who were aware of their BP levels, possessed higher KCVDs. This suggests that AoBP should be included in public or educational campaigns related to CVDs. Currently, in South Korea, the first comprehensive plan for CVD management (2018–2022), including improvement of public awareness of CVDs’ warning signs, has been established to reduce, prevent, and manage CVDs. In the integrated management model for chronic diseases including HTN [60], it is necessary to include the importance of AoBP in counseling and education for hypertensive patients, and to check whether they measure and are aware of their blood pressure in the evaluation stage. Furthermore, public campaigns and regular policy support for improving CVD warning signs including AoBP are required. Among the factors associated with KCVDs in our study, living alone and low education were also reported to delay CVD treatment in other studies. Therefore, education/public campaigns and policy support are important for people living alone or with a low education level so that they recognize CVDs signs and visit the hospital through emergency calls and ambulance services. Furthermore, the result of this study can serve as basic data for developing socio-cultural interventions, aimed at mitigating CVDs, by improving levels of KCVDs.

## Figures and Tables

**Table 1:** General characteristics according to blood pressure level awareness.

Variables	Categories	Awareness Group (*n* = 17,039)	Non-Awareness Group (*n* = 12,793)
		*n* (Column %) or M ± SD	*n* (Column %) or M ± SD
Age		65.99 ± 10.441	72.84 ± 10.483
	19–39	155 (0.9)	65 (0.5)
	40–64	7230 (42.4)	2526 (19.7)
	Over 65 years old	9564 (56.7)	10,202 (79.7)
Education	<High school	11,518 (67.7)	11,240 (88.0)
	High school or higher	5491 (32.3)	1538 (12.0)
Monthly income (Korean won)	<2 million	8512 (51.6)	8556 (69.0)
	≥2 million	7999 (48.4)	3843 (31.0)
Employed	Yes	7369 (43.3)	4263 (33.3)
	No	9653 (56.7)	8522 (66.7)
Residence location	Rural (town, township)	8168 (47.9)	8221 (64.3)
	Urban (city)	8871 (52.1)	4572 (35.7)
Marital status	Married and living with a spouse	11,087 (65.2)	5902 (46.2)
	Other *	5930 (34.8)	6885 (53.8)
Smoking status	Non-smoker	16,708 (98.1)	12,455(97.4)
	Current smoker	331 (1.9)	338 (2.6)
Alcohol (during the last year)	Yes	7971 (46.8)	4580 (35.8)
	No	9068 (53.2)	8212 (64.2)
BMI	Normal	6882 (40.4)	5953 (46.5)
	Obese	10,157 (59.6)	6840 (53.5)
Walking exercise	<30 min, 1 day per week or less	5010 (29.4)	5486 (42.9)
	>30 min, 2–4 days per week	3517 (20.6)	2584 (20.2)
	>30 min, 5 days per week or more	8512 (50.5)	4723 (36.9)
HTN medication	Yes	16,428 (96.4)	12,351 (96.6)
	No	610 (3.6)	440 (3.4)
Diabetes Mellitus	Yes	3895 (22.9)	3233 (25.3)
	No	13,140 (77.1)	9558 (74.7)

Data are presented as number (%) or mean ± SD; * divorced, separated, widowed, or never married; BMI: body mass index; HTN: hypertension; M ± SD: mean ± standard deviation.

**Table 2:** KCVDs and psychological factors according to blood pressure level awareness.

Variables	Awareness Group (*n* = 17,039)	Non-Awareness Group (*n* = 12,793)	*t* or χ^2^	*p*	Cohen’s d or Cramer’s V
	M ± SD or *n* (%)	M ± SD or *n* (%)			
Subjective health status	2.76 ± 0.85	2.50 ± 0.90	−15.443	<0.001	0.29
Depression	2.69 ± 3.65	3.42 ± 4.28	−9.752	<0.001	−0.19
HRQoL	0.88 ± 0.152	0.82 ± 0.196	24.700	<0.001	0.35
Good KCVDs	10,140 (64.3)	5635 (35.7)	701.172	<0.001	0.15
Poor KCVDs	6899 (49.1)	7158 (50.98)			

Data are presented as number (%) or mean ± SD; statistical significance *p* < 0.05; abbreviations: HRQoL: health-related quality of life; KCVDs: knowledge on warning signs of cardio-cerebrovascular diseases.

**Table 3:** Factors associated with knowledge of the cardio-cerebrovascular disease.

Variables	Categories	OR	95% CI		*p*
			LL	UL	
Age	19–39	0.976	0.840	1.133	0.747
	40–64	1.046	1.012	1.081	0.008
	65–79	ref.			
Education	<High school	0.931	0.899	0.964	0.000
	High school or higher	ref.			
Monthly income (Korean won)	<2 million	0.989	0.961	1.019	0.464
	≥2 million	ref.			
Employed	Yes	1.034	1.006	1.063	0.017
	No	ref.			
Residence location	Rural (town, township)	1.005	0.978	1.033	0.714
	Urban (city)	ref.			
Marital status	Married and living with a spouse	1.068	1.039	1.097	0.000
	Other *	ref.			
Smoking status	Non-smoker	0.993	0.914	1.080	0.875
	Current smoker	ref.			
Alcohol (during the last year)	No	0.983	0.957	1.009	0.196
	Yes	ref.			
BMI	Normal	0.987	0.962	1.012	0.302
	Obese	ref.			
Walking exercise	<30 min, 1 day per week or less	0.964	0.936	0.993	0.015
	>30 min, 2–4 days per week	0.987	0.955	1.021	0.454
	>30 min, 5 days per week or more	ref.			
HTN medication	Yes	0.974	0.910	1.042	0.442
	No	ref			
Diabetes Mellitus	Yes	1.010	0.981	1.041	0.488
	No	ref.			
AoBP	Yes	1.121	1.091	1.151	0.000
	No	ref.			
Subjective health status		1.000	0.983	1.016	0.972
Depression		1.000	0.997	1.004	0.799
HRQoL		1.073	0.979	1.175	0.132

It was multiple univariate analysis; statistical significance *p* < 0.05; * divorced, separated, widowed, or never married; abbreviation: BMI: body mass index; HTN: hypertension; AoBP: awareness of own BP level; HRQoL: health-related quality of life.

**Table 4:** Factors associated with blood pressure level awareness.

Variables	Categories	OR	95% CI		*p*
			LL	UL	
Age	19–39	1.092	0.795	1.500	0.587
	40–64	1.595	1.491	1.706	<0.001
	65–79	1			
Education	<High school	0.542	0.503	0.584	<0.001
	High school or higher	1			
Monthly income (Korean won)	<2 million	0.969	0.914	1.028	0.297
	≥2 million	1			
Employed	Yes	1.111	1.050	1.176	<0.001
	No	1			
Residence location	Rural (town, township)	0.637	0.603	0.673	<0.001
	Urban (city)	1			
Marital status	Married and living with a spouse	1.496	1.418	1.578	<0.001
	Other *	1			
Smoking status	Non-smoker	1.162	0.984	1.373	0.078
	Current smoker	1			
Alcohol (during the last year)	No	0.956	0.905	1.008	0.097
	Yes	1			
BMI	Normal	0.832	0.790	0.875	<0.001
	Obese	1			
Walking exercise	<30 min, 1 day per week or less	0.736	0.693	0.781	<0.001
	>30 min, 2–4 days per week	0.898	0.839	0.960	0.002
	>30 min, 5 days per week or more	1			
HTN medication	Yes	1.437	1.249	1.653	<0.001
	No	1			
Diabetes Mellitus	Yes	1.024	0.965	1.087	0.426
	No	1			
Subjective health status		1.095	1.059	1.132	<0.001
Depression		0.994	0.987	1.001	0.088
HRQoL		2.048	1.704	2.462	<0.001
Knowledge of CVDs		1.076	1.068	1.085	<0.001

It was multiple univariate analysis; statistical significance *p* < 0.05; * divorced, separated, or widowed or never married; abbreviation: BMI: body mass index; HTN: hypertension; AoBP: awareness of own BP level; HRQoL: health-related quality of life.

## Data Availability

Not applicable.
